# Understanding the Impact of the Nurse Manager’s Vocation for Leadership on the Healthcare Workplace Environments in Mexico: A Grounded Theory Approach

**DOI:** 10.3390/nursrep14020093

**Published:** 2024-05-15

**Authors:** Angeles Yañez-Lozano, Manuel Lillo-Crespo

**Affiliations:** Administration of Health Institutions, University Institute of Hispanic Nations, Pachuca de Soto 42084, Mexico; manuel.lillo@iunhi.edu.mx

**Keywords:** vocations, leadership, work environment, nursing

## Abstract

Background: Leadership in any managerial position that a nurse may hold appears to be closely connected to fostering positive and productive work environments within healthcare settings. However, not all nurse managers are characterized by leadership, and not all nurse leaders are nurse managers. In countries, such as Mexico, those who occupy these roles have barely sufficient training in management, are mainly characterized by their experience in one clinical specialty and their vocation for leadership is not a requirement. Our study aims to understand how the leadership vocation that some nurse managers have in their daily practice could impact the work environments of healthcare organizations in Mexico. Methods: A qualitative design was carried out through a grounded theory approach based on Corbin & Strauss, interviewing individually 13 nurse managers with representative experience in Mexican public sector hospitals. Results: According to the interviewees, the meaning of vocation is based mainly on the love for their profession, on the vocation of being a nurse and caring for people, as well as on the development of their own natural leadership potential for decision-making in practice. This situation produces positive feedback on themselves by generating productive effects in the work environment, consequently strengthening them to better organize professional resources and therefore producing improvements. Conclusions: The nurse manager’s vocation of leadership derives from both the vocation of being a practicing nurse and the vocation for being a leader, positively impacting and productively contributing to the improvement of the work environment. Consequently, nurse managers with vocation for leadership should be the gold standard role in any healthcare organization. This study was retrospectively registered with the (registros-OSF-bmyvz-v1) on the (26 July 2023) registration number (10.17605/OSF.IO/BMYVZ).

## 1. Introduction

In recent times, much has been discussed about the need for professional roles, specifically trained or with natural skills, in management and leadership for core decision-making positions in healthcare organizations, mainly in periods of healthcare crisis and in situations of economic deficit affecting countries’ healthcare systems, such as the recent situation experienced with the COVID-19 pandemic worldwide. However, little has been published on the recommendable profiles for nurse managers who play a crucial role in day-to-day decision-making at healthcare organizations, especially in countries where nurses are not highly socially recognized. In real-life scenarios, most of these positions are occupied by professionals with extensive healthcare experience, although not necessarily in management, sometimes these roles may also be filled by people with other affinities not always having the essential managerial competencies required [1]. There are few cases in which management skills, leadership potential, and training in this field are taken into account, and even fewer cases where a selection process takes place encouraging candidates to expose a management project that should be evaluated by experts in the field and monitored in practice [2]. Moreover, there are few validated instruments and tools for evaluating the performance of these managers. Additionally, some authors directly relate this situation to the social image, the gender orientation, and the level of academic and research development achieved by the nursing profession in some contexts and nations [3].

One of the critical questions that often arise concerning leadership vocation and management positions in healthcare and nursing practice is related to the essence and nature of leadership in the nurse managers’ positions and how these aspects impact the workplace environment of healthcare organizations; indirectly affecting the quality of care provided to the attended population. If nursing care work in practice has been primarily considered vocational [4], it would also be interesting to explore whether the vocation to lead and the competence to manage such care, the human and material resources used in clinical settings, and the nursing staff is also vocational; therefore, prompting an inquiry into whether there exists a relationship between nursing leadership vocation and the orientation of nurse managers’.

Therefore, is it truly necessary that nurses in their role as managers (also understood as head of nurses, nursing administrators, chief nursing officers, directors, supervisors, coordinators, and managers, among others) are to be driven by the vocation to lead for better results of their management? Is having the vocation to be a nurse and caring for others sufficient to become a nurse manager and consequently positively influence the work environment? Can leadership be trained? or is it necessary to have, in addition to the vocation to care, the capacity to manage and the vocation to lead? To answer these questions, we must first define these key terms: vocation, leadership, and workplace environment. 

### 1.1. Vocation and Nursing

Initially, authors such as Gallard [5] and Ponto [6] stated that nursing vocation is closely aligned with professional practice, centered on those values that lead to internalizing the procedure and acting with awareness, knowledge, and a sense of belonging; in other words, the professional nursing vocation becomes an individual precept, [7,8]. In this sense, according to Hernández [7], vocation is an inner voice that leads towards the profession and the exercise of a certain activity; it is considered the tendency towards any status, career, or profession [6,7,8]; at a general level, vocation appears related to desires and to what is inspiring for each person, which is consistent with preferences, interests and aptitudes, knowledge and skills [9]. It is also considered a process that is developed throughout life, as it is permanently constructed, being aware that it is not a process that ends over the years, as it evolves and consolidates throughout the human process [10]. It seems then that this vocation in the field of health and specifically in nursing care contributes to improve the discipline and promotes the development of professional models characterized for being effective, appropriate, human, proactive and person-centered, for the population attended and the professional itself; all with adequate communication and an identity feeling. In this sense, vocation contains a call and a response from the professional, the need to meet the others in their decisive events in life. 

### 1.2. Leadership and Nursing

Secondly, Antonakis [11] defines leadership as a formal or informal process that takes place between a leader and a group of followers. This process depends on the leader’s traits and behaviors, as well as on the followers’ attributions of the leader’s characteristics, made in relation to the results of the leader’s actions [12]; the description of the nurse itself, in its role as a leader, and the attributions of its followers in relation to its actions; in the administrative part is understood by organizing, directing exclusively [13,14], a situation that is not far from the nursing profession. In fact, in many contexts, we find people who are leaders without being managers and the other way around. In line with this, it seems that leadership has to do with an inner part, such as the vocation to do something like caring for others; while management competencies could be trained without that inner part. Thus, someone could be educated to become a nurse without having vocation for caring as well as someone could be educated in managerial competencies without having vocation for leading. There are managerial styles (which is the way of exercising power) that correspond to leaders while others do not [15], and this is something that also happens in the Nursing profession. Furthermore, it has been highlighted that the behavior of the nurse in its role as manager may be determined by the leadership style used daily [16], and is likely to implement practices in operational resources, which boost its leadership style [17]. 

Depending on their orientation, there are specific leadership models, depending on the personal characteristics of the managers. For instance, in autocratic and democratic leadership, the former is rigid and inflexible in management, making decisions unilaterally and limiting employee participation; the latter involves employees in decision-making and encourages the decision of work methods and objectives [18]. The liberal leadership or participative style consists of absolute freedom of action of the group members [19]; the transactional leadership style, in which goals are achieved by implying the possibility of direct gains (sense of achievement, recognition, remuneration) and losses [17]. The transformational or oriented leadership style offers the possibility of transformation to subordinates, inspiring them to perform better than they originally intended [20]; The formative style is very effective, offering autonomy to the team to set their own goals, providing guidance to the employee to promote their professional development [21]. In addition, leadership plays a crucial role in strengthening organizations, including those dedicated to health [22].

### 1.3. Workplace Environment and Nursing

Workplace environment is conceptualized as the psychological environment resulting from behaviors, management models and organizational policies, which is reflected in interpersonal relationships [23], it is known to be an element that is part of the organizational culture, but not only that, it also exists between conditions and work climate, where a perception is generated in the workers that can influence their behavior [24], such as leadership and vocation in the case of the nurse manager, by combining both elements within the organizational management process, as it affects the workers and consequently is reflected in the efficiency, effectiveness, warmth and quality of care that a patient receives, within the health organizations [25,26]. Leadership and organizational environment may be related aspects that positively or negatively affect the well-being of companies, organizations, and workers. The skills of a leader are essential to generate healthy, good, or positive workplace environments that foster the growth and development of subordinates [27]. Studies show that most of the organizational climate that exists in a company is influenced by the leadership style of its manager [28]. Consequently, it has been documented that an unfavorable or negative climate tends to decrease both quality of work life and intellectual capital and job satisfaction [29], which significantly impacts job performance and well-being at work [30,31] and thus negatively impacts population health and outcomes. It seems logical that a vocational leader would foster a more positive work environment. 

So far, the evidence in the literature has shown that the aspects to be studied have been approached from independent quantitative paradigms, with respect to the work environment, studying health organizations [32] about the type of leadership with management activities [33] regarding vocation, its associated factors have been studied from the practice of nursing in students [34] and teacher training [35], also from the perspective of caring, but not from a professional perspective. Given this situation, a lack of knowledge exists concerning vocation, regarding leadership in nursing management functions and the work environment; denoting the importance of studying this topic, to understand its meaning in depth, as well as, to value these concepts in the current world that society lives in, specially from a context-based perspective. 

Based on the above, it is necessary to qualitatively analyze and understand the impact of leadership vocation on the nurse manager and, in turn, its repercussions on the workplace environment of health organizations. For such purpose, we chose Mexico which is a country where women mostly perform the nursing profession. Their professional competencies’ regulations are nowadays still being developed, with reduced areas of autonomous practice, limitations in academic education, high salary, and professional practice variabilities, and high dependency on other health professionals especially in terms of managerial positions. 

Nurses in Mexico do not have officially regulated training in managerial competencies during their undergraduate studies, as happens in other countries, few are postgraduate programs that do, they have not developed their academic careers to the maximum until yet, and their position in the national health organizations’ management schedule and hierarchy are not equivalent to other health professionals, normally depending on the medical directors. Moreover, the health system in Mexico is very bureaucratic with logistical failures, with little citizenship participation, given this situation, and the conditions described above, we seek to know what motivates nurses to perform their leadership management functions and how this impacts the work environment. 

### 1.4. Hypothesis and Objectives

We aim to explore and understand the meaning of managerial vocation and leadership in a nursing management position, and its impact on the healthcare work environment in Mexico. Specifically, we seek to identify influential socio-demographic characteristics, such as gender, years of experience, and academic background. We aimed to determine whether these factors are associated with leadership vocation in nurse managers within the work environment, as well as to explore its significance and describe its impact on the workplace environment.

## 2. Materials and Methods

### 2.1. Study Design and Techniques

A qualitative study was conducted through a grounded theory approach based on the method of Corbin and Strauss using in-depth interviews. This approach is suitable for developing concepts and frameworks to represent participants’ experiences and to explore complex and latent social processes and patterns [36]. Grounded theory is a systematic methodology that involves the construction of theories through methodological collection and analysis of data [36]. This method was chosen because it allows researchers to use data to actively respond to a given phenomenon and ultimately generate a theory that provides a map of action for a given situation [37]; in this case, in nurse managers, the sense of vocation in leadership was analyzed and related to its influence and impact on the work environment.

### 2.2. Population, Sample, and Sampling

The study population was the nurse managers working in one regional hospital in Tlaxcala, Mexico in the public sector. Hospital clinical settings were considered the most representative environments belonging to second-level care hospitals from the public sector, given that in the different services they perform management functions, coordination of human resources, and provide care; these activities are related to the variables that will be studied and, for such reason, community nursing, district nursing, and primary care nursing were left out. Participants were recruited through theoretical sampling, which involves decisions about where to optimally find data for an emerging theory [38]. Eighteen interviews were planned in the design and conducted, though data saturation occurred in the 13th interview. The interviewees were intended to be representative, experienced in nursing management, having contributed to improvements within their clinical environments, and willing to provide feedback on the topic. The inclusion criteria were as follows:

- Have a minimum of 5 years’ experience as a manager in their working environment.
- Have at least some professional training, specialization, and/or postgraduate degree in nursing.
- Currently holding the position of nurse manager (understood as any formally designated management responsibility).

These criteria were used to ensure the representativeness of the sample, looking for nurse managers who showed intrinsic characteristics of leadership and management; with these characteristics, in the Mexican health system, a nurse can safely exercise the management functions within the hospital institutions.

A total of 18 nurses who met the inclusion criteria were accepted to participate, with the following characteristics: 4 heads of nurses, who only performed managerial and administrative functions, and 14 nurse managers (from the Intensive Care Unit, Emergency, Operating Theatre, and Internal Medicine), who were linked both to care and managerial functions. Regarding the work shift, 14 out of the total were in the morning shift and 4 were in the evening shift; this proportion corresponded to reality and all of them were women, because in Mexico there are usually more women in those positions. Only 3 head nurses refused to participate when invited in the beginning, citing being busy with their activities, although we sought them out on repeated occasions.

To collect data about vocation, leadership, and work environment, semi-structured interviews were used to obtain a greater domain on the topic to be investigated. In this sense, according to Taylor and Bogdan [39], the interview consists of repeated face-to-face encounters between the interviewer and the informants, aimed at understanding the informants’ perspectives on their lives, experiences, and situations, as expressed in their own words. In this type of interview, the researcher is the research instrument itself; with the help of a pre-created script of questions, data about socio-demographic characteristics and inclusion criteria were gathered, e.g., What is your academic background? How long have you been working in the institution? Have you taken any management courses? What about vocation, leadership, and work environment, for example, do you know what a vocation is? How do you define a management profession? Do you think that a vocation profession is necessary to be a nurse manager? Does vocation in management help you to develop leadership characteristics? Do you consider yourself a nurse manager with vocation and leadership? What kind of leader do you consider yourself as a nurse manager? Do you think that vocation and leadership influence the work environment? How do you consider the work environment, from your situation as a nurse manager? To have a deeper understanding of the topics, good and bad practices (understood as what works and what does not work) in their workplace were also discussed, so they could mention examples of each of those situations.

### 2.3. Ethical Considerations

The present study complied with the provisions of the General Health Law on Health Research [40], specifically concerning article 14, sections VII and VIII, approved by the State Committee for Health Research of the Tlaxcala, Mexico Ministry of Health with registration number CDCyES-OIyC-CEIS-001-2023, and with the approval of the Research Ethics Committee with registration number CEI-23022023. In addition, informed consents were obtained from the participants. For the security of the participants’ responses, individualized devices were used for each participant, assigning them a code as the recording was finished; a new device was used for the next participant, where only the researcher played the audio once the data collection was finished, outside the data collection site.

### 2.4. Data Collection and Analysis

The data collection was carried out in an orderly and organized manner, during March and April 2023; each interview lasted approximately 70 min, and the total number of minutes recorded was 380. Pilot tests were conducted so that the researcher was trained and had a clearer picture of the participants. Electronic equipment was prepared to capture each of the interviews, quiet spaces to work were chosen, respecting the privacy of the participants, where only the researcher was with the subject of study. For data collection, consent was first requested to begin recording; in an atmosphere of trust, the questions were asked to the participants, allowing them to talk about the subject.

The data collected from the interviews were transcribed into Word format, for the analysis of the information, using hand-coded coding to search for the core category, which is the main construct of the research [41,42], using the grounded theory approach based on the method of Corbin and Strauss with open and axial coding.

Data processing was performed by the researcher, the themes identified were derived from the data collected, and no software was used for data analysis. The data were processed manually. Each participant was assigned a code from 1 to 13, according to the sample size. The analysis technique was content analysis. Arguments were contrasted to bring out the empirical categories. This was carried out in three phases and was verified manually and individually.

An oscillatory reading was made of the arguments contrasted during the pre-analysis phase to conceive the original impressions. The colorimetric method was used to select the recording units. For the phase of exploration of the material, an in-depth reading of the textual body was considered indispensable; the data that emerged were processed and unified in units that favored the representation of the characteristics appropriate to the content of the discourses. The register units that emerged were marked with different colors and the nuclei of meaning that originated the categories and subcategories were sought.

For the exploration of the material, an in-depth reading of the textual body was carried out: the initial data were transformed, organized, and gathered into units that allowed the description of the relevant characteristics of the content; the register units that emerged were identified with different colors, and the core of meaning that gave rise to the categories was sought. To develop the phase-of-results processing, inference, and interpretation, it was necessary to carry out the categorical analysis: the frequency weighted to the categories that resulted from the analysis was used, which favored the verification of the frequency weight of each category. The main researcher carried out the coding of the data.

A cross-triangulation analysis was then carried out by three researchers with experience in this type of analysis, outlining explanations of the phenomenon, and the data was analyzed until theoretical saturation was found. During the data analysis process, the research team met to discuss coding and to help organize the core phenomenon as well as the categories (Table 1).

### 2.5. Rigor and Reliability

To ensure the scientific rigor of the results, the following interventions were made in the interviews:

- The researcher returned to the informants twice during the data collection process to confirm the findings and review specific data. This was done to verify information that resulted in findings acknowledged by the informants, drawing from their experiences, feelings, and thoughts.
- In the selection of informants, specific criteria were applied, focusing exclusively on nurse managers in the hospital environment. Simultaneously, the literature search was adjusted to the management context.
- The interviews were transcribed word-for-word to maintain the veracity of the answers. The analysis of the information was carried out by the “Principal Investigator” and then shown to two other expert investigators, to assure the same or similar conclusions, who had similar perspectives.
- At the end of the analysis of the information, a triangulation with two expert researchers on the subject was sought, to visualize the reality found in the information, sharing the perspective of the researchers, based on the information.

## 3. Results

Of the 18 interviews, data saturation was reached after the 13th interview, when the repetition of the participants’ responses began to be observed, the emergence of the same codes as the information was collected, and different information was no longer found about the topic, with the answers being repetitive, as the participants were free to express everything related to the topic. The participants provided socio-demographic data before starting the interviews (Table 2). The participants were considered representative, with experience in the practice of health management, and willing to give their opinion on the topic. All of them were recommended by other professionals, who acted as key informants, following a snowball sampling process.

The socio-demographic characteristics of the participants are presented in Table 2, including the academic training of the participants, the postgraduate training, the years of working life in the institution, the area where they work, and the type of contract.

### Presentation of the Comprehensive Model

**Figure 1:**
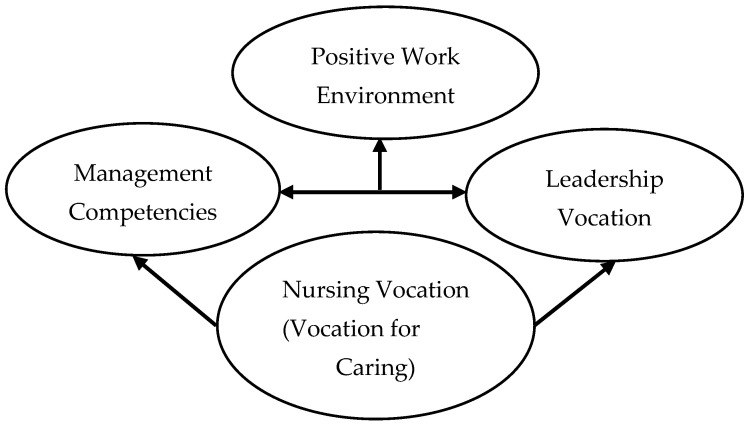
Comprehensive model on the influence and impact of the nurse manager’s leadership vocation on the health care work environment in Mexico.

In the comprehensive theoretical model developed through our grounded theory approach, (Figure 1) it was identified that from the practical vocation of nursing to care of people (central category), the managerial vocation and the vocation for leadership (both asso-ciated categories), considered by the respondents as different sorts of vocation, emanate. There was consensus in the responses of all the participants on the fact that the practical vocation of nursing determines both the managerial vocation and the leadership, pre-senting a relationship between both concepts however the interviewees referred to them as different things.

The first associated category is the "managerial vocation" which appears with dif-ferent reasons for the performance of these managerial functions and competencies though it is not considered as “being a leader”. The second associated category is "Leadership", the participants referred to different types, and that could be increased with knowledge and constant training. Unlike the managerial vocation, Leadership is always identified with the capacity to generate positive work environments. Therefore, the nurse manager’s capability to create positive workplace environments derives from the practical vocation of nursing to care for people and the vocation for leadership ac-cording to our model.

The rationale and main assumptions of this model are:

As for the meaning of the nursing vocation (vocation for caring) (our core category), it was expressed by all the participants “as doing something they like to do”, and “love for the profession”. With this, it can be deduced that vocation could come from something deeper, (something that is born) which helps to develop the vocation of the nurse manager.

P1: (…) “Vocation is something we like to do”.

P5: (…) “Well, I think that vocation is born, since you like your career, you like what you do, what you do” (…) so well, it helps you to become a manager.

P3: (…) “I think that vocation is what we like to do, and we are on the way to doing it (…) ”well, to share that vocation that one feels for, for the discipline or knowledge…

P6: (…) “I think that whoever has a vocation is because they love their profession (…) “I think that in management I think we should like it since we are in contact with patients and staff too”.

In the interview, the participants referred to the nursing vocation (vocation for caring) (central category) as it helps to develop leadership vocation (associated category). With this, it can be deduced that the vocation for leadership could come from something deeper, which naturally helps to develop leadership in the nurse manager:

P2: (…) “Vocation, if it helps us, it helps us, but being a leader is also more difficult because if we do not know how to be a good leader, we cannot manage and make decisions well”.

P4: (…) “Of course, to have that vocation you have to own leadership”.

P9: (…) “Vocation surely helps, yes. Why do I think so? Because, we come back to the same thing, if you don’t have passion for what you do, if it doesn’t come from an inner part of you, you limit yourself. And when you have passion, you have an extra value”.

Participants commented on the importance of “leadership vocation” to have a positive work environment:

P2: (…) “Look, it’s complicated. But I think that, when we have leadership, and being in front with the different colleagues and seeing their needs, we can make good teamwork, working with a nice working environment”.

P3: (…) “Well, I think that, if I didn’t have the power of leadership, it would be very difficult to work with the amount of people or human resources that we work with, right? And to fear a good working environment, then leadership is fundamental”.

About the work environment, the Mexican nurses described it as adequate, since being such different people, they organize themselves to provide a service in patient care, saying that talking about different situations makes them work better together to find solutions to the different situations that arise:

P1: (…) “Well, I define it as we are, we are organized, we have a good working team, we work together. I think it’s good, it’s good”.

P2: (…) “It’s good, I always think that maybe they see some details that need to be discussed in the group, but it’s always good”.

P3: (…) “It’s a lot, it’s very nice, we really work as a team, and the staff is very cooperative and available”.

P4: (…) “It’s very good! good. We all have to have responsibility, this tranquility, now yes, it’s a very good climate”.

## 4. Discussion

In this article, we seek to prioritize the understanding of the influence and impact of the nurse manager’s leadership vocation in healthcare work environments in Mexico. As mentioned in the results, the vocation to be a nurse is a central axis for the performance of managerial role and vocation for leadership, and the participants stated that the latter is the one that helps to develop a good working environment. However, when comparing the concepts of vocation, management, leadership, and working environment, the literature can could be confusing, and this situation is crucial as more studies are needed to analyze the interaction of these concepts, moving towards identifying, selecting, and training the best profiles to lead professional teams in a way that could generate the best workplace environments possible. 

In our results, the nursing practical vocation is linked to the managerial role and the vocation for leadership in nursing professional practice, and this relates to Gallard [5] who stated that vocation is a process that integrates the link between a person and their social, political, and geographical context. In line with this, our informants also concluded that a nurse manager able to influencing and impact positively its workplace environment and teamwork, should be boosted by the nursing professional vocation to care, the managerial competencies and skills trained along its career and its vocation for leadership. Our findings are also similar with the idea that managerial role contributes to managing the workforce, even to influence them, to do a good job, thus leadership is an inner part, the ability to take the initiative to lead, gather, promote, encourage, motivate, be inclusive, and evaluate a group or team [43]. 

Our results are also coincident with two studies conducted in China and the Sultanate of Oman stating that leadership has significant direct effects on nurses’ work environment [44] and that leaders’ competencies and charisma achieve organizational goals and lead the organization to excellence [2]. It is documented that the work climate is adequate, in the literature is reported as a healthy climate, which is the perception of nurse managers [44]. Therefore, our comprehensive model allows the understanding of the influence and impact of the nurse manager’s leadership vocation on healthcare work environments in Mexico, identifying the fundamental role of practical vocation in staff in managerial roles.

### Limitations

Although we selected the most representative clinical settings available within our reach, and chose in each of them the professionals who could contribute the most according to the type of sampling, the objective of the study, the inclusion criteria, and their representativeness; there were many variations in the characteristics of the informants, such as the years of experience as nurse managers, that might have resulted in a quite high variability of the participants’ subjectivity and therefore this might have influenced the final interpretation. On the contrary, the lack of some clinical settings in the representation of the participating nurse managers’ current contexts may or may not have contributed to leave out other perceptions and views that might have enriched our outcomes and illuminated even more the understanding of the phenomenon. Finally, our results do not show the voices and experiences of all nurse managers in the country, from all potential contexts in Mexico, and therefore future research approaches would benefit from the inclusion of more representative participants from other workplaces to demonstrate whether the model we are presenting potentially suits other cases. 

## 5. Conclusions

Our study presents a model that comprehensively explains how Mexican nurse managers recognize the positive influence of having a vocation not only for the Nursing profession but also for leadership in their daily functions related with patient care, team management and in the exercise of the rest of managerial functions; and how both vocations seem to improve the workplace environment. In addition, we have understood that the nurse manager’s vocation for caring and leading are factors that positively in-fluence the workplace environment as they increase the nurse managers’ willing to keep learning more about management competencies, enhancing the performance of work activities and especially aiding in the management of the nursing workforce and the interprofessional teamwork. Furthermore, our findings highlight the aspects that should be encouraged at different levels of the nursing training curricula and continuing professional development to identify and promote the adequate profiles of the future nurse managers that will consequently improve the workplace environments and generate greater autonomy and confidence in the daily practice of the nursing workforce.

## Figures and Tables

**Table 1:** Central phenomenon, categories, and subcategories.

Phenomenon	Category
Management development	Nursing Vocation (Vocation for Caring)
	Management Competencies
Leadership and management generation	Leadership Vocation
Leadership and the work environment	Positive Work Environment

Note: authors’ design.

**Table 2:** Interviewee profiles.

Participant	Gender	Basic Training	Postgraduate Training	Professional Experience	Work Area	Type of Contract
1	Female	Bachelor of Nursing	Master of Nursing Services Administration	16 years	Emergency	Permanent
2	Female	Bachelor of Nursing	Master in Public Health	16 years	Emergency	Permanent
3	Female	Bachelor of Nursing	Master in Public Health	17 years	Operating Theatre	Permanent
4	Female	Bachelor of Nursing and Midwifery	Master’s Degree in Administration of Health Institutions	18 years	Intensive Care Unit	Permanent
5	Female	Bachelor of Nursing	-----	15 years	Internal Medicine	Permanent
6	Female	Bachelor of Nursing	Skill in administration	37 years		Permanent
7	Female	Bachelor of Nursing	-----	18 years	Intensive Care Unit	Permanent
8	Female	Bachelor of Nursing	Surgical Nursing Specialist	36 years	Internal Medicine	Permanent
9	Female	Bachelor of Nursing	Master in Administration of Nursing Services	27 years	Emergency	Permanent
10	Female	Bachelor of Nursing	-----	37 years	Operating Theatre	Permanent
11	Female	Bachelor of Nursing	Surgical Nursing Specialist	30 years	Internal Medicine	Permanent
12	Female	Bachelor of Nursing	Master in Administration of Nursing Services	25 years	Emergency	Permanent
13	Female	Bachelor of Nursing	-----	17 years	Operating Theatre	Permanent
14	Female	Bachelor of Nursing	Master in Public Health	16 years	Emergency	Permanent
15	Female	Bachelor of Nursing	Master in Public Health	17 years	Operating Theatre	Permanent
16	Female	Bachelor of Nursing	-----	18 years	Intensive Care Unit	Permanent
17	Female	Bachelor of Nursing	Surgical Nursing Specialist	36 years	Internal Medicine	Permanent
18	Female	Bachelor of Nursing	Master in Administration of Nursing Services	27 years	Emergency	Permanent

Note: authors’ design.

## Data Availability

Data available in a publicly accessible repository with DOI https://doi.org/10.17605/OSF.IO/BMYVZ. Publicly available datasets were analyzed in this study. This data can be found here: https://archive.org/details/osf-registrations-bmyvz-v1, accessed on 23 April 2024.

## Public Involvement Statement

No public involvement in any aspect of this research.

## Guidelines and Standards Statement

This manuscript was drafted against the reporting of qualitative research studies and O’Brien BC, Harris IB, Beckman TJ, Reed DA, Cook DA., standards for reporting qualitative research: a synthesis of recommendations. Acad Med. 2014;89(9):1245–1251 or qualitative research.
